# The Role of Prior HBV Infection on the Efficacy of 3TC/DTG as a Maintenance Therapy

**DOI:** 10.3390/v18010142

**Published:** 2026-01-22

**Authors:** Tommaso Matucci, Sara Occhineri, Alessandra Palomba, Maria Linda Vatteroni, Laura Del Bono, Marina Polidori, Riccardo Iapoce, Alberto Borghetti, Marco Falcone

**Affiliations:** 1 Infectious Diseases Unit, Department of Clinical and Experimental Medicine, Azienda Ospedaliero Universitaria Pisana, University of Pisa, 56122 Pisa, Italy; tommaso.matucci@unifi.it (T.M.);; 2 Virology Unit, Azienda Ospedaliera Universitaria Pisana, University of Pisa, 56122 Pisa, Italy

**Keywords:** HIV infection, antiretroviral therapy, occult HBV infection, dual ART, virological failure

## Abstract

Lamivudine/dolutegravir (3TC/DTG) is an effective and well-tolerated antiretroviral regimen for most people with HIV (PWH) who are virologically suppressed; however, specific clinical characteristics, such as prior hepatitis B virus (HBV) exposure or archived resistance-associated mutations (RAMs), may influence the risk of virological failure (VF). We conducted a retrospective, monocentric cohort study to evaluate the incidence and predictors of VF among PWH who switched to 3TC/DTG after achieving virological suppression (HIV-RNA < 50 copies/mL). A total of 188 PWH were included. Over 5082 patient-years of follow-up (PYFU), 8 individuals (4.3%) experienced VF, corresponding to an incidence rate of 1.45 per 1000 PYFU. The cumulative probabilities of VF at 1, 2, 3, 4, and 5 years were 0.6%, 2.7%, 2.7%, 4.2%, and 22.3%, respectively. In exploratory multivariable analyses, anti-HBc positivity was associated with an increased risk of VF (adjusted hazard ratio [aHR] 4.80, 95% CI 1.03–22.43; *p* = 0.046). After adjustment for age and sex, individuals with anti-HBc positivity who had switched from a tenofovir-containing regimen showed the highest risk of VF compared with anti-HBc-negative individuals without prior tenofovir exposure (aHR 15.06, 95% CI 1.40–161.38; *p* = 0.025). Given the limited number of virological events, these findings should be interpreted with caution. Nevertheless, they suggest that prior HBV exposure, particularly in the context of tenofovir discontinuation, may represent a clinically relevant factor when considering simplification to 3TC/DTG.

## 1. Introduction

Dual antiretroviral therapy (ART) with dolutegravir (DTG) and lamivudine (3TC) represents an effective and well-tolerated treatment option both as first-line therapy and as a simplification strategy for People living with HIV (PWH) [1]. Nevertheless, several limitations have been identified by international treatment guidelines [1,2]. In ART-naïve individuals, contraindications include high baseline viral load and prior failure of tenofovir/emtricitabine-based pre-exposure prophylaxis (PrEP). In treatment-experienced patients, the use of 3TC/DTG is generally recommended in the absence of resistance to nucleoside reverse transcriptase inhibitors (NRTIs) or integrase strand transfer inhibitors (INSTIs) [1,2]. Recent updates to the European AIDS Clinical Society (EACS) guidelines have expanded the potential use of this regimen to individuals with archived M184V mutations, provided that virological suppression is maintained [1]. Conversely, current European and North American guidelines for the management of HIV [1,2] and hepatitis B virus (HBV) infection [3,4,5] advise against the use of dual therapy in the presence of active HBV infection, defined by HBsAg positivity. The interaction between HIV and HBV is well documented and is associated with poorer immunological recovery, increased immune activation, and higher rates of virological failure and liver-related complications [6,7,8,9]. Although isolated anti-HBc positivity is not considered an absolute contraindication to dual therapy, emerging evidence suggests that this serological profile may also be associated with inferior virological outcomes with 3TC/DTG [6]. The aim of the present study was therefore to evaluate the incidence and predictors of virological failure in a real-world cohort of PWH treated with 3TC/DTG, with particular focus on the role of prior HBV exposure as assessed by anti-HBc serostatus.

## 2. Materials and Methods

We conducted a retrospective cohort study at a tertiary referral university hospital in Pisa, Italy. Adult (≥18 years) PWH were eligible if they were receiving stable ART and switched to 3TC/DTG after achieving virological suppression, defined as HIV-RNA < 50 copies/mL in at least one determination prior to the switch. Eligible switches occurring between 1 January 2015 and 31 December 2023 were included. Exclusion criteria were positive HBsAg serostatus at baseline, prior exposure to 3TC/DTG, and the absence of at least one post-switch HIV-RNA determination. Baseline was defined as the date of switch to 3TC/DTG.

The primary endpoint was time to virological failure (VF), defined as the earliest occurrence of one of the following events: (a) the first of two consecutive HIV-RNA values > 50 copies/mL; (b) a single HIV-RNA ≥ 200 copies/mL, or (c) a single HIV-RNA between 50 and 200 copies/mL followed by treatment discontinuation or intensification. Participants were censored at treatment discontinuation (TD) not meeting VF criteria, at the date of last available viral load measurement or loss-to-follow-up (defined as the absence of any HIV-RNA determination for more than 12 months). HIV-RNA determinations were performed according to routine clinical practice.

Probability of time to VF was assessed using Kaplan–Meier estimates. Predictors of VF were evaluated using Cox proportional hazards models: variables associated with the outcome at univariable analysis (*p* < 0.05) were included in a multivariable Cox regression model. Potential predictors of VF included demographic characteristics (age, gender, risk factor for HIV acquisition), HIV-related variables (time since HIV diagnosis, time of continuous virological suppression at BL, zenith HIV-RNA, nadir and BL CD4 count, residual viremia at BL, genotypic susceptibility score for lamivudine based on historical genotype and calculated with the Stanford algorithm, version 9.8) and antiretroviral therapy prior to switch. The role of previous HBV infection, defined by the presence of anti-HBc antibodies in the absence of HBsAg positivity, was also investigated. Missing data were limited for most variables, except for resistance-associated mutations, which were unavailable for approximately 27% of participants due to the absence of historical genotypic resistance testing. Given the non-random nature of this missingness, multiple imputation was not performed. Instead, for the multivariable Cox regression, a sensitivity analysis was conducted excluding individuals with missing genotypic data or with documented lamivudine resistance mutations (*n* = 5).

A post hoc analysis was conducted to assess whether occult HBV infection had a causal role in time to VF. Differences in the demographic and viro-immunological characteristics of PWH with and without anti-HBcAg positivity were assessed using the Chi-square test for categorical variables and Student’s t-test for continuous variables, to explore potential confounders of anti-HBcAg serostatus. A multivariable Cox regression model was fitted to assess the independent role of anti-HBc positivity on VF (potential confounders were chosen among variables that showed a statistically different distribution in the population with positive and negative anti-HBcAg serostatus, at a *p*-value < 0.05).

Clinical and treatment-related information was retrospectively collected from paper-based medical records. Laboratory data, including HIV-RNA measurements and immunological parameters, were obtained from the institutional electronic laboratory database. All analyses were performed with Stata Statistical Software (StataCorp, 2017. Stata Statistical Software: Release 15. College Station, TX, USA: StataCorp LLC).

## 3. Results

A total of 188 patients were included in our study: most were male (141, 75%), Caucasian (174, 92.5%), and with a median age of 54 years (IQR 44–61 years). Sexual transmission was the most common route of HIV acquisition, with similar proportions of individuals reporting heterosexual (HET) (70, 37.2%) and same-sex sexual contacts (84, 44.7%). The median time since HIV diagnosis was 11 years (IQR 5–17 years), with a median of 9 years since ART initiation (IQR 5–16 years), and 5 years of virological suppression (IQR 3–9). Thirty-five PWH (18.6%) had a history of a previous AIDS-defining condition. Complete characteristics of the study population are summarized in Table 1.

The reason for the shift to dual therapy was a proactive switch in all cases, with 129 (68.6%) patients switching from a 2NRTIs + INSTI regimen. One hundred and twenty-eight PWH (68.1%) had experienced at least one previous VF, and 127 (67.6%) were previously exposed to five or fewer regimens.

Among participants with at least one genotypic resistance test available before BL (137, 72.9%), 14 (10.1%) had at least one RAM to NRTIs; RAMs to 3TC were detected in 5 (3.6%) participants, including one case (0.7%) with an M184V mutation.

Concerning HBV serostatus, most patients were HBV-seronegative (74, 39.4%). Fifty-one (27.1%) had isolated anti-HBsAg positivity, and 35 (18.6%) were positive for both anti-HBsAg and anti-HBcAg. Ten patients (5.3%) had isolated positivity for anti-HBcAg, and 18 (9.6%) had an unknown HBV serostatus.

Nineteen (10.1%) patients had a positive HCV serostatus.

Sixteen patients discontinued treatment with 3TC/DTG after a median time of 27 months (2.87 per 1000 patient-years of follow-up or PYFU). Reasons for TD included the following: VF (6/16, 37.5%), switch to a long-acting regimen (2/16, 12.5%), drug-related toxicity (5/16, 31.3%), and other/unknown causes (3/16, 18.7%).

**Figure 1:**
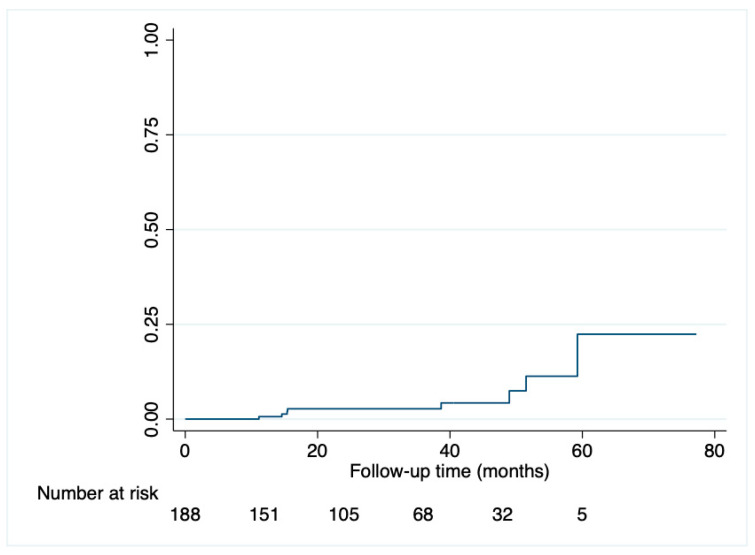
Kaplan–Meier-estimated probability of virological failure over time.

VF occurred in eight patients (1.45 per 1000 PYFU) (Figure 1).

The estimated probability of VF was 0.6% (95% CI 0.1–0.4) at 12 months, 2.7% (95% CI 1.0–7.1) at 24 and 36 months, 4.2% (95% CI 1.7–10.7) at 48 months, and 22.3% (95% CI 7.9–54.3) at 60 months (Figure 1).

Factors associated with VF at univariable analysis were as follows: baseline HIV-RNA between 20 and 49 copies/mL (versus < 20 copies/mL, HR 5.67, 95% CI 1.10–9.39, *p* = 0.039), a higher genotypic susceptibility score (based on Stanford algorithm, version 9.7) for 3TC (per 10-point increase, HR 1.74, 95% CI 1.23–2.48, *p* = 0.002), and positive anti-HBcAg serostatus (versus negative, HR 5.76, 95% CI 1.26–26.24; *p* = 0.024). Given the low number of events, a multivariable Cox model was fitted only including anti-HBc serostatus (positive versus negative, aHR 4.80, 95% CI 1.03–22.43, *p* = 0.046) and baseline HIV-RNA (20 and 49 copies/mL versus < 20 copies/mL, aHR 5.27, 95% CI 0.81–34.37, *p* = 0.082). As a sensitivity analysis, we excluded people with resistance mutations to 3TC (5 persons, with only one case of previously detected M184V) and with unknown resistance history (129 people were included in the final model, with 5 VFs): anti-HBcAg positivity was still associated with increased risk of VF at multivariable analysis (aHR 13.21, 95% CI 1.35–129.37; *p* = 0.027).

Table 2 summarizes the associations among other potential predictors and the virological outcome (Table 2).

Among PWH with and without anti-HBcAg-positive serostatus, gender and age were significantly different, with a higher proportion of older men in the anti-HBcAg-positive group. Interestingly, fewer PWH in the anti-HBcAg-positive group switched from a tenofovir-containing strategy (see Table 3).

After stratifying anti-HBcAg serostatus by prior tenofovir exposure, we found a 15-fold higher risk of VF in PWH with anti-HBcAg positivity and previous exposure to tenofovir (versus negative anti-HBcAg and no prior tenofovir use, aHR 15.06, 95% CI 1.40–161.38; *p*-value = 0.025) (Table 4). The effect of anti-HBcAg positivity was markedly reduced in those not switching from a tenofovir-based therapy and did not reach statistical significance (Table 4).

## 4. Discussion

The dual ART regimen with 3TC/DTG proved highly effective in our cohort of virologically suppressed PWH undergoing ART optimization. The most recent European guidelines [1] highlight the possibility of switching to 3TC/DTG in individuals with prior VF and/or in the presence of M184I/V mutations. Regarding HBV serological status, switching is recommended in the presence of anti-HBs antibodies, although no absolute contraindications are currently provided for isolated anti-HBcAg positivity [1,2].

In our study, anti-HBcAg positivity was associated with a higher risk of VF. Moreover, we found a 15-fold higher risk for VF among those anti-HBcAg seropositive who discontinued tenofovir.

To date, no clear evidence has demonstrated an increased risk of VF with 3TC/DTG in the context of prior HBV infection. However, a large study from the Italian ICONA cohort by Malagnino et al. reported an excess of risk of VF in individuals with isolated anti-HBcAg positivity, regardless of ART regimen, although this risk remained lower than in those with active HBV infection [7]. In another analysis by the same group, significantly fewer anti-HBcAg-positive PWH achieved target undetected HIV-RNA levels, compared with anti-HBcAg-negative individuals, after switching to 3TC/DTG; moreover, anti-HBcAg positivity was the only factor associated with suboptimal HIV suppression [10]. Conversely, a similar study conducted in China involving 601 PWH switching to 3TC/DTG found no differences in the proportion of PWH with undetectable viremia after 24 months [11]. Finally, in another Italian cohort of 606 virologically suppressed PWH switching to 3TC/DTG, no significant differences by HBV serostatus were observed in the risk of VF or viral blips; however, the effect of HBV serology was not adjusted for potential confounders [12].

Another unresolved issue concerns the lack of a confirmed biological mechanism explaining the reduced efficacy of ART in the setting of prior HBV exposure. Recently, cryptic serum HBV-DNA replication was demonstrated in a cohort of anti-HBc-positive/HBsAg-negative PWH despite ongoing tenofovir exposure [13]. After switching to a tenofovir-sparing regimen (mostly 3TC-based), the proportion of PWH with HBV-DNA>10 IU/mL increased from 12.9% at T1 to 42.6% at T2 and was predicted by a lower nadir CD4 count and the presence of cryptic HBV-DNA at baseline. Although HIV virological failure was not specifically reported during follow-up, these findings support a previously proposed hypothesis [10,14] that suggested a synergistic viral interplay between HIV and HBV coinfection, whereby reduced antiviral drug pressure or the development of HBV resistance to 3TC may allow HBV rebound or low-level replication which in turn could promote HIV transcription through HBx activity [14].

The relevance of archived resistance associated mutations (RAMs) to 3TC in influencing the effectiveness of 3TC/DTG is still debated due to conflicting data [15,16,17,18,19,20,21,22,23]. In the present study, a higher risk of VF was also predicted by the presence of 3TC-associated RAMs in previous genotypic tests, although such mutations were overall rare, with M184V being reported in only one case. This topic has been the subject of extensive debate in recent years, with no definite evidence of a causal effect of the main 3TC resistance mutation, M184I/V, on virological outcomes. In the pilot ART-PRO clinical trial, no increased risk of VF was observed at week 144 in PWH with prior M184I/V, provided that the mutation was absent in the HIV-DNA genotypic test at screening [24]. More recently, the SOLAR-3D clinical trial, which included participants with and without a history of M184I/V (some with detectable mutations at baseline HIV-DNA testing), confirmed the absence of differences in rates of virological suppression and viral rebound at week 144 [25]. In contrast, one retrospective study reported that M184I/V increased the risk of viral rebound, particularly when combined with at least one thymidine analogue mutation (TAM), regardless of the duration of prior virological suppression [26]. Moreover, an emulated trial from the Italian ARCA cohort found a higher risk of failure with the dual regimen when the switch occurred within six months of virological suppression and in the presence of historical RAMs (either TAMs or isolated M184I/V), with a non-significant trend towards superior efficacy of triple therapy in this context [20]. Despite these discrepancies, which may partly reflect differences in study populations and methodologies, it is not possible to completely rule out an effect of historical RAMs on the efficacy of dual therapy. Caution therefore remains advisable when considering this strategy in individuals with a history of virological failure, even though the overall risk of failure, especially with resistance development, remains very low [27].

Our study has several limitations, primarily related to the low incidence of virological outcomes that makes the predictive model potentially overfitted, even if a parsimonious multivariable Cox model was chosen. The retrospective design also limited the possibility to capture relevant data, such as adherence, and to assess HBV-DNA at baseline and at failure, which could have clarified the role of occult HBV infection as the cause of VF. Finally, the monocentric design limits the generalizability of study results that should therefore be taken with caution.

Despite these limitations, our study partly confirms previous findings regarding the potential impact of prior HBV infection when switching to tenofovir-sparing regimens. Considering that most next-generation treatment strategies will lack HBV activity, further research on this topic is warranted.

## 5. Conclusions

We found that 3TC/DTG was safe and effective, in line with the available literature. However, positive anti-HBcAg serostatus was particularly relevant in influencing virological failure, especially in individuals switching from a tenofovir-based regimen. Despite the limitations of our study, we believe that careful consideration of other specific viro-immunological characteristics (e.g., duration of viral suppression, persistence of RAMs over time) remains essential when planning optimization to 3TC/DTG in the context of previous HBV infection.

## Figures and Tables

**Table 1:** Characteristics of study population at baseline.

Population	*N* = 188 (% or IQR)
Male gender (%)	141 (75.0)
Age, years (IQR)	54 (44–61)
Ethnicity (%)	
- Caucasians	174 (92.5)
- Africa-Sub-Saharan	4 (2.1)
- South America	7 (3.7)
- Asians	3 (1.6)
HIV acquisition, risk factor (%)	
- Heterosexual men and women	70 (37.2)
- Men who have sex with men	84 (44.7)
- People who inject drugs	14 (7.5)
- Other/unknown	20 (10.6)
Time since HIV diagnosis, years (IQR)	11 (5–17)
Time since ART initiation, years (IQR)	9 (5–16)
Time of continuous virological suppression, years (IQR)	5 (3–9)
Previous AIDS event, at least one (%)	35 (18.6)
Nadir CD4+ count, cells/μL (IQR)	270 (144–385)
Baseline CD4+ count, cells/μL (IQR)	716 (538–920)
Zenith HIV-RNA levels (%):	
- <100.000 copies/mL	86 (47.2)
- 100.000 –499.999 copies/mL	54 (29.7)
- ≥500.000 copies/mL	42 (23.1)
Baseline HIV-RNA levels (%):	
- Target not detected	110 (58.5)
- Target detected < 20 copies/mL	62 (33.0)
- 20–49 copies/mL	16 (8.5)
Positive HCV-Ab serostatus (%)	19 (10.1)
Previous regimen (%)	
- 2NRTI + INSTI	129 (69)
- 2NRTI + NNRTI	39 (21)
- 2NRTI + PI	6 (3)
- Other dual regimen	12 (6)
- Not specified	2 (1)
Number of previous therapeutic lines (IQR)	4 (3–6)

Abbreviations: IQR, interquartile range; ART: antiretroviral therapy; NRTI, nucleoside reverse transcriptase inhibitor; INSTI, integrase strand transfer inhibitor; NNRTI, non-nucleoside reverse transcriptase inhibitor; PI, protease inhibitor.

**Table 2:** Cox regression: predictors of time to virological failure.

	HR (95% CI)	*p*-Value	aHR (95% CI)	*p*-Value
Age (per 10 years more)	1.02 (0.54–1.91)	0.953	-	-
Sex (female vs. male)	0.44 (0.05–3.60)	0.440	-	-
Nadir CD4 count	0.99 (0.99–1.00)	0.110	-	-
Zenith HIV-RNA	0.99 (0.99–1.00)	0.607	-	-
Years with HIV	1.00 (0.92–1.08)	0.919	-	-
Years of virological suppression	0.88 (0.72–1.07)	0.205	-	-
Baseline HIV-RNA 20–49 copies/mL (versus <20 copies/mL)	5.67 (1.10–9.39)	**0.039**	5.27 (0.81–34.37)	0.082
GSS-3TC (per 10 points more)	1.74 (1.23–2.48)	**0.002**		
Pre-switch tenofovir exposure	1.87 (0.44–7.84)	0.395	-	-
Anti-HBcAg+ (vs. negative)	5.76 (1.26–26.24)	**0.024**	4.80 (1.03–22.43)	**0.046**
HBV serology:				
-Anti-HBcAg-/Anti-HBsAg-	Ref	Ref	-	-
-Anti-HBcAg-/Anti-HBsAg+	1.43 (0.41–5.02)	0.574	-	-
-Anti-HBcAg+/Anti-HBsAg+	1.71 (0.41–7.23)	0.466	-	-
-Anti-HBcAg+/Anti-HBsAg-	2.69 (0.31–3.30)	0.368	-	-

Note: Values in bold refer to statistically significant associations. The multivariable model only included baseline HIV-RNA and anti-HBcAg serostatus, due to the low incidence of the outcome. Abbreviations: (a) HR, (adjusted) hazard ratio; CI, confidence interval; GSS, genotypic susceptibility score; 3TC, lamivudine.

**Table 3:** Population characteristics and risk of virological failure according to anti-HBcAg serostatus.

	Anti-HBcAg+ *n* = 45 (%)	Anti-HBcAg- *n* = 135 (%)	*p*-Value
Sex (male)	41 (91.1)	95 (70.3)	**0.005**
Age (years, IQR)	58 (55–61)	52 (50–54)	**0.002**
Risk factor for HIV acquisition			0.120
-HET men/women	11 (24.4)	55 (40.7)	
-MSM	23 (28.1)	59 (43.7)	
-IDUs	3 (6.6)	10 (7.4)	
-Other/Unknown	8 (1.7)	11 (8.1)	
Years with HIV (IQR)	14.43 (11.64–17.23)	11.74 (10.31–13.18)	0.072
Years of suppression (IQR)	7.27 (5.37–9.18)	5.85 (5.09–6.62)	0.101
CD4 baseline (IQR)	644 (493–857)	734 (548–947)	0.109
HIV-RNA detectable (20–49 copies/mL)	4 (8.8)	11 (8.1)	0.987
Pre-switch Tenofovir exposure	15 (33.3)	72 (53.3)	**0.020**
Previous virological failure	13 (28.8)	32 (23.7)	0.651
3TC resistance associated mutations	2 (4.4)	3 (2.2)	0.695

Abbreviations: IQR, interquartile range; HET, heterosexual; MSM, men who have sex with men; IDUs, intravenous drug users; 3TC, lamivudine.

**Table 4:** Cox regression for risk of virological failure per anti-HBcAg serostatus and previous tenofovir exposure.

	aHR (95% CI)	*p*-Value
Previous tenofovir use and occult infection:		
- No prior tenofovir plus anti-HBcAg–	Ref	Ref
- Prior tenofovir plus anti-HBcAg–	1.51 (0.13–16.92)	0.738
- No prior tenofovir plus anti-HBcAg+	2.62 (0.14–47.41)	0.513
- Prior tenofovir plus anti-HBcAg+	**15.06 (1.40–161.38)**	**0.025**
Age (per 10 years more)	0.93 (0.43–2.04)	0.859
Sex (female vs. male)	NA *	NA *

Note: * Hazard ratios and confidence intervals not computable, due to absence of VFs among women. Abbreviations: aHR, adjusted hazard ratio; CI, confidence interval.

## Data Availability

The original contributions presented in this study are included in the article. Further inquiries can be directed to the corresponding author.

## Abbreviations

The following abbreviations are used in this manuscript:

HIV	Human immunodeficiency virus
HBV	Hepatitis B virus
3TC	Lamivudine
3TC/DTG	Lamivudine/dolutegravir
PWH	People living with HIV
ART	Antiretroviral therapy
VF	Virological failure
RAMs	Resistance associated mutations
OBI	Occult HBV infection
TD	Treatment discontinuation
PYFU	Patient year of follow-up
HR	Hazard ratio
CI	Confidence interval
GSS	Genotypic susceptibility score
PrEP	Pre-exposure prophylaxis
MSM	Males who have sex with males
HCV	Hepatitis C virus
IDUs	Intravenous drug users
NRTI	Nucleoside reverse transcriptase inhibitor
INSTI	Integrase strand transfer inhibitor.
